# Mechanical Properties and Deformation Mechanisms of Nanocrystalline U-10Mo Alloys by Molecular Dynamics Simulation

**DOI:** 10.3390/ma16134618

**Published:** 2023-06-27

**Authors:** Xuelian Ou, Yanxin Shen, Yue Yang, Zhenjiang You, Peng Wang, Yexin Yang, Xiaofeng Tian

**Affiliations:** 1 The College of Nuclear Technology and Automation Engineering, Chengdu University of Technology, Chengdu 610059, China; 2 Center for Sustainable Energy and Resources, Edith Cowan University, Joondalup, WA 6027, Australia; 3 Applied Nuclear Technology in Geosciences Key Laboratory of Sichuan Province, Chengdu University of Technology, Chengdu 610059, China

**Keywords:** mechanical properties, deformation mechanism, molecular dynamics, U-Mo alloy, deformation twinning, phase transition

## Abstract

U-Mo alloys were considered to be the most promising candidates for high-density nuclear fuel. The uniaxial tensile behavior of nanocrystalline U-10Mo alloys with average grain sizes of 8–23 nm was systematically studied by molecular dynamics (MD) simulation, mainly focusing on the influence of average grain size on the mechanical properties and deformation mechanisms. The results show that Young’s modulus, yield strength and ultimate tensile strength follow as average grain size increases. During the deformation process, localized phase transitions were observed in samples. Grain boundary sliding and grain rotation, as well as twinning, dominated the deformation in the smaller and larger grain sizes samples, respectively. Increased grain size led to greater localized shear deformation, resulting in greater stress drop. Additionally, we elucidated the effects of temperature and strain rate on tensile behavior and found that lower temperatures and higher strain rates not only facilitated the twinning tendency but also favored the occurrence of phase transitions in samples. Results from this research could provide guidance for the design and optimization of U-10Mo alloys materials.

## 1. Introduction

Uranium (U) is the primary component of nuclear fuels. The Reduced Enrichment for Research and Test Reactors (RERTR) Program began to advance in the late 1970s, which primarily aimed to replace highly enriched uranium (HEU) fuels in reactors with low enriched uranium (LEU) fuels, thereby reducing the use of nuclear materials and the risk of proliferation [1,2,3,4]. Higher fuel density is required at lower uranium enrichment levels to keep reactors performing. The most potential candidate for high-density fuels is considered to be U-Mo alloy [5,6,7,8,9,10], which exhibits much higher uranium density and thermal conductivity than traditional fuels used in reactors, as well as lower neutron capture cross-section, better irradiation behavior and tolerable swelling reaction. Adding molybdenum (Mo) stabilizes the high-temperature cubic γ-U phase, which enhances its irradiation behavior, such as dimensional stability [11]. Furthermore, the increase in molybdenum concentration leads to higher mechanical stability [12]. Uranium alloy containing 10 wt% molybdenum (U-10 wt.% Mo) has attracted attention from researchers due to its small thermal expansion coefficient and high thermal conductivity [13,14,15,16].

Compared with coarse-grained materials, nanocrystalline (NC) metals exhibit much higher strength or hardness, enhanced toughness, decreased elastic modulus and ductility, providing great value for applications in engineering materials and structures, becoming the impetus for extensive research [17,18,19]. Grain refinement leading to polycrystalline metals with novel and unique mechanical properties has been widely reported. According to Kim et al. [20], both ductility and elastic modulus reduced with decreasing grain size. Tensile tests on NC Zn were performed by Zhang et al. [21] at room temperature, and they found that the smaller the grain size, the greater the yield strength, but the worse the ductility. In addition, the Hall–Petch effect was weakened or even destroyed when the grain size of nanocrystals was smaller than their critical grain size [22,23]. Hence, it is necessary to establish a scaling law between the mean grain size and mechanical parameters in NC materials.

In metals and alloys, deformation twinning and dislocation slip were recognized as two main and competing modes of plastic deformation [24]. Strain rate and temperature considerably impact the tendency of deformation twinning. It was proved that higher strain rates and lower temperatures facilitate the formation of deformation twinning in nanomaterials [25,26,27]. The ductility of nanomaterials can be increased or decreased by twinning. Abundant twin boundaries can act as preexisting dislocation nucleation positions, thus enhancing the ductility of structures with relatively few slip systems [28]. Conversely, deformation twins may cause the destruction of grain boundaries, thus reducing the extensibility of the material [29,30,31].

Artificial defects are difficult to avoid in the preparation of nanomaterials, and the above defects mask the inherent mechanical properties of materials. Consequently, molecular dynamics (MD) simulation has evolved into a powerful tool for exploring their mechanical characteristics and deformation mechanisms and has been widely used in nanoscale studies. Thus far, MD simulations have offered important insights into the deformation mechanisms of nanocrystalline metals. Our previous research on Mo [32] showed that the combination of grain rotation and twinning could promote grain coalescence and growth and improve the ductility in small-sized grains. The simulation results of the tensile process for NC Ni by Van Swygenhoven et al. [33,34] showed that movements such as grain boundaries slip and grain rotation occurred during the deformation process without cracks. The grain boundaries sliding and dislocations emission dominate the plasticity deformation of smaller and larger-sized grains, respectively. Pan et al. [35] observed that stress-induced phase transitions from the bcc structure to fcc and hcp structure occurred locally during the deformation of NC Ta, and the hcp structure was derived from the fcc structure. Fang et al. [36] found that for NC Cu, grain rotation caused grain growth, and strain hardening behavior was only observed in small-sized grains. Yamakov et al. [37] investigated the complex interactions between dislocation and grain boundary process during the plastic deformation of NC Al and revealed three twinning nucleation mechanisms, including heterogeneous and homogeneous nucleation.

However, the fundamental mechanical properties and deformation mechanisms of NC U-10Mo alloys have rarely been using MD simulations, yet this is much needed. In this study, polycrystalline U-10Mo alloy models with average grain sizes of 8–23 nm were established. We carried out MD simulations on the uniaxial stretching of samples to investigate their mechanical characteristics and deformation patterns during tension, taking into account the effects of grain size, temperature and strain rate. Additionally, atomic displacement and local shear strain were also investigated. These simulation results can offer theoretical guidance for the material design and engineering application of U-10Mo alloys, aiming to improve their properties and expand their applications. The article is organized as follows: Section 2 introduces our simulation method and model. Section 3 presents and thoroughly discusses the simulation results. Conclusions are given in Section 4.

## 2. Simulation Method and Model

**Figure 1:**
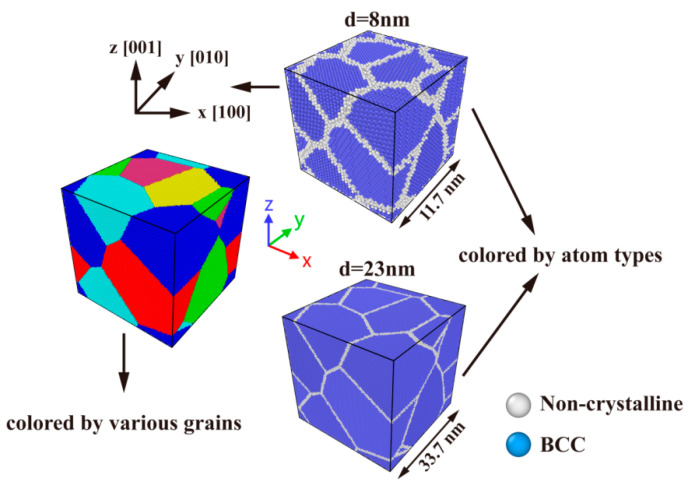
Atomic illustrations of polycrystalline U-10Mo alloys with different grain sizes of *d* = 8 nm and *d* = 23 nm. Uniaxial tensile loading is applied in the X-direction.

To determine how the mechanical characteristics and deformation mechanisms of NC U-10Mo alloys were affected by grain size, a series of periodic 3D polycrystalline models with various sizes were constructed using the Atomsk code [38] based on the Voronoi construction method [39]. The crystal unit was a body-centered cubic (bcc) structure, and its orientations were x-[100], y-[010] and z-[001]. Periodic boundary conditions (PBC) were applied in three directions to eliminate boundary effects. All samples were made up of six grains, but the average grain size changed from 8 nm to 23 nm. The correlation between the number of grains *n* and the average grain size *d* was given by the following equation [40]:

(1) $$n=\left[\frac{6V}{\pi d^{3}}\right]$$

where *V* is the volume of the simulated sample, and the notation [ ] indicates round. The simulation systems’ lengths changed between 11.7168 and 33.6858 nm to regulate the average grain size. Each grain’s geometrical characteristics and orientation were kept constant, thereby obtaining self-similar samples. The number of atoms in simulated systems ranged from 67,770 to 1,697,944 as the mean grain size increased. Detailed information on the initial atomic models is provided in Supplementary Table S1. The initial atomic structures of NC U-10Mo alloys with average grain sizes *d* = 8 and 23 nm are exhibited in Figure 1. Notably, U-10Mo alloy in this study was expressed by mass percentage; that is, U-10Mo alloy represented U-10 wt.% Mo alloy.

The interaction between atoms was described using Angular-Dependent Potential (ADP) for U-Mo, which was recently developed by Starikov et al. [41]. The Newtonian motion equation was integrated using the Verlet-velocity integration algorithm [42] with a 0.001 ps time step. Before loading, all simulated models were completely balanced for 100 ps at 300 K temperature and zero pressure in the NPT ensemble. By this balancing, the system would have enough time to obtain an appropriate locally balanced structure. The system’s temperature and pressure were calibrated using a Nose/Hoover thermostat [43] and a Nose/Hoover barostat [44], respectively. After equilibrium, the uniaxial stretching load was imposed by slightly scaling in the x-direction at every timestep, and the maximum elongation in the x-direction was 50%. During tensile deformation, the strain rate exerted on the sample was 5 × 10^8^ s^−1^. Moreover, the y- and z-direction can move flexibly to keep zero pressure. Throughout the deformation procedure, the temperature was maintained at room temperature. The common neighbor analysis (CNA) method [45,46] was used to distinguish atomic structure as well as to study defects evolution during stretching processes. All simulations were carried out via the widely used open-source software package LAMMPS [47], and all atomic configuration images in this study were exported using the visualization tool OVITO [48].

## 3. Results and Discussion

### 3.1. Scaling Laws of Mechanical Properties

**Figure 2:**
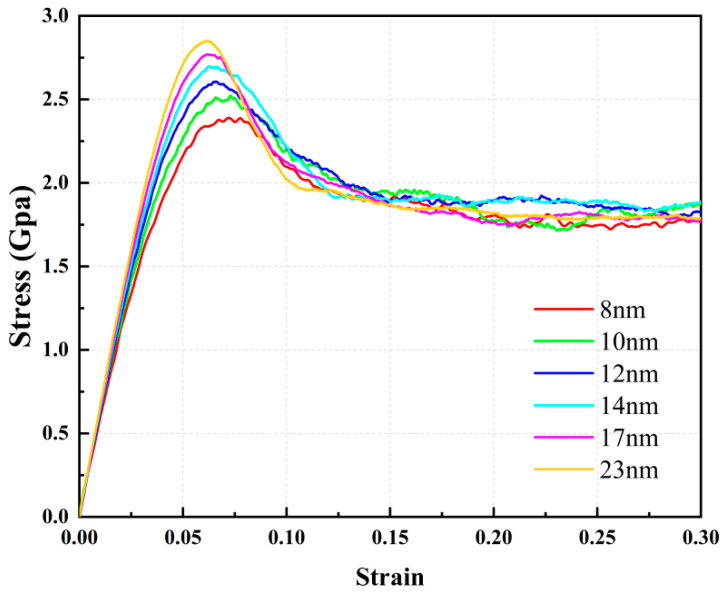
Uniaxial tensile stress–strain curves of polycrystalline U-10Mo alloys with various mean grain sizes of 8, 10, 12, 14, 17 and 23 nm at 300 K.

Figure 2 provides predicted stress–strain curves for NC U-10Mo alloys with average grain sizes of 8, 10, 12, 14, 17 and 23 nm. For simplicity, only six representative curves are displayed. The stress–strain curves of all simulated samples are supplied in Figure S1 of the Supplementary Materials. It was observed that all stress–strain curves showed analogous trends and could be clearly distinguished into three distinct phases of deformation: (1) Polycrystalline materials exhibit linear elastic behavior during the initial deformation stage. (2) As strain increases, materials yielding and non-linear plastic behavior is observed until samples reach ultimate tensile strength (UTS). (3) After reaching UTS, there is a stress drop in the curve, followed by the materials entering a uniform plastic flow area, showing excellent overall plasticity. This sudden stress drop has often been observed in nanocrystals experiments due to the perfusion nucleation and proliferation of defects in perfect nanocrystals after yielding. Interestingly, for larger-sized samples, the stress reduction following peak stress appears to be more significant, which is discussed later.

From stress–strain curves, several typical mechanical parameters were extracted for investigation. Young’s modulus *E* is acquired by fitting the curve slope over a strain range of 0% to 2%, and yield strength *σ*_s_ is obtained from the stress of 0.002 offset strain, defining UTS *σ*_b_ as the peak stress in the curve. Detailed mechanical parameters for all tested polycrystalline U-10Mo alloys at 300 K can be found in Table S1 of the Supplementary Materials.

**Figure 3:**
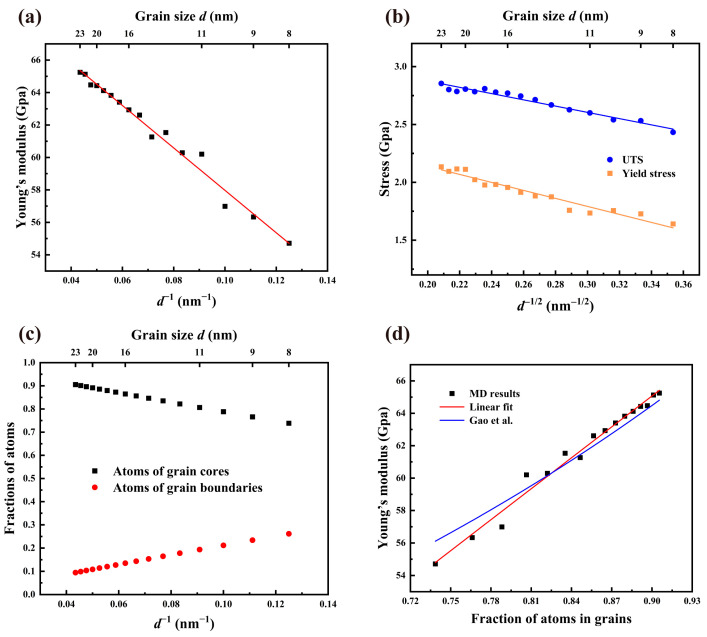
(**a**) Relationship between Young’s modulus *E* at 300 K and the reciprocal of mean grain size *d*^−1^ of polycrystalline U-10Mo alloys. (**b**) Variations in yield strength and UTS with the reciprocal of the square root of the mean grain size *d*^−1/2^. (**c**) Evolution of fractions of atoms in grain cores and grain boundaries with the reciprocal of mean grain size *d*^−1^. (**d**) Relationship between Young’s modulus and fraction of atoms in grain cores.

Abundant investigations on conventional polycrystalline metals have disclosed the dependence of Young’s modulus on grain size. For instance, the linear correlation between *E* and *d* for nanocrystals was reported by Nan et al. [49]. Previous experiments and MD simulations have observed the proportionate relationship between *E* and *d*^−1^ in conventional metals, such as Mo [50], Pt [51] and Cu [52]. In order to disclose the similar correlation in NC U-10Mo alloys, Figure 3a displays Young’s modulus *E* for NC U-10Mo alloys with various average grain sizes. As can be seen, the *E* value rises as the average grain size increases. For instance, when *d* rises from 8 nm to 23 nm, *E* improves by 19.3%, from 54.71 GPa to 65.25 GPa, agreeing with the experimental value of 65 GPa by Ozaltun et al. [1]. Furthermore, we observed that *E* and *d*^−1^ have a nearly linear relationship in accordance with the following formula:

(2) $$E=71.047-130.739d^{-1}$$

The linear model’s R-squared (R^2^) value is 0.9822, indicating that it closely approximates the outcomes of our MD simulation.

After the size-dependence of Young’s modulus has been determined, the relationship between average grain size and strength, which includes yield strength and UTS, is focused on. Figure 3b shows the variation in yield strength *σ*_s_ and UTS *σ*_b_ as *d*^−1/2^, respectively. With growing average grain size, both mechanical parameters rise monotonically. As *d* changes from 8 nm to 23 nm, for instance, the yield strength and UTS increase from 1.64 and 3.06 to 2.13 and 4.20, representing an increase of 29.9% and 37.3%, respectively. Notably, the relationship between two parameters and *d*^−1/2^ verifies an inverse Hall–Petch effect; that is, the strength reduces as grain size decreases. This phenomenon is further supported by the tensile stress–strain curves of all samples (refer to Figure S1). Similar inverse Hall–Petch effect has been found when the average grain size of nanocrystalline metals and alloys is below their critical size [51,53,54].

As is well known, since the atomic arrangement density within the grains is often greater than that on the grain boundaries, the number of bonding atoms within the grains is larger than that at the grain boundaries, such that the binding energy within the grains is higher than that at the grain boundaries, which leads to the higher Young’s modulus of the atoms in the grains compared with the atoms at the grain boundaries. It has directive significance to explore the influence of the fraction of atoms in grains on Young’s modulus. Figure 3c shows the variation in the fraction of atoms in grains *φ_g_* and grain boundaries *φ_gb_* with *d*^−1^, respectively. We observed that with increasing average grain size, the *φ_gb_* value reduces while the *φ_g_* value increases. Moreover, *φ_gb_* and *φ_g_* are nearly perfectly linear with *d*^−1^.

Young’s modulus *E* is shown in Figure 3d as a function of the fraction of atoms in grains *φ_g_*. Likewise, there is a linear relationship between *E* and *φ_g_*. According to previous investigations focusing on polycrystalline metals, Young’s modulus of grain boundary cannot achieve a third of that grain [52]. Based on a linear constitutive theory, Gao et al. [55] proposed a formula predicting Young’s modulus of nanocrystals:

(3) $$E=\frac{1}{\frac{\varphi_{g}}{E_{core}}+\frac{\varphi_{gb}}{E_{GB}}}$$

where *E* is Young’s modulus of polycrystalline samples, and *E_core_* and *E_GB_* are Young’s modulus of grain core and GBs components, respectively. Assuming the fraction of atoms in grain cores is 1, Equation (2) is evolved into *E* = *E_core_*. Then we can fit the relationship between Young’s modulus *E* and the fraction of atoms in grains *φ_g_* by Equation (3), as demonstrated by the blue line in Figure 3d. The fitting result shows that Young’s modulus of grain boundary is about 34.8 GPa, which is about 48.9% of that of the grain, 71.049 GPa. According to Figure 3d, it seems that the linear fit is better to describe the relationship between *E* and *φ_g_* than Equation (3). The values of R^2^ for linear fit and Equation (3) are 0.98115 and 0.94621, respectively, confirming the observation. In summary, the conclusion can be drawn that the proportion of atoms in grains improves as the average grain size increases, resulting in a higher Young’s modulus for NC U-10Mo alloys, consistent with previous results obtained by simulations [36,50,56,57]. We can believe that the fundamental reason for the significant increase in Young’s modulus with grain size is the larger fraction of atoms in grains, which supports the higher Young’s modulus. Moreover, this can be regarded as an essential characteristic of nanocrystals when the grain size is only dozens of nanometers.

### 3.2. Deformation Processes

**Figure 4:**
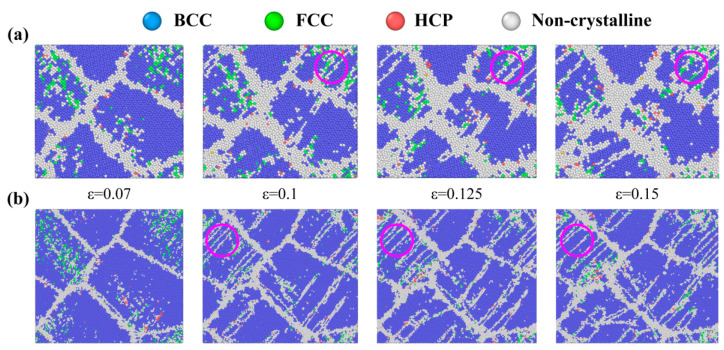
Snapshots of atomic configurations of NC U-10Mo alloys with the mean grain size of (**a**) 11 nm and (**b**) 23 nm at different tensile strains, deformed at 300 K and a strain rate of 5 × 10^8^ s^−1^. Atoms are colored according to the structure determined by CNA. Blue, green, red and white atoms correspond to BCC, FCC, HCP and other structures, respectively.

In order to investigate the deformation mechanism and microstructure evolution of polycrystalline U-10Mo alloys, the atomic structures of two typical samples with *d* = 11 and 23 nm, which, respectively, represent small and large grain sizes, were selected for comparison, as illustrated in Figure 4. Grain boundary migration was observed at 7% strain. Following the yield point, twins begin to appear to release stress. The twinning embryo is initially formed by [111]/6 shearing dislocations on (112) planes, occurring at the grain boundary. Twins are mainly nucleated by the continuous emission of Shockley partials from the grain boundaries. Subsequently, the twin bands are blocked by the opposite grain boundaries and thickened by layer-by-layer lateral parallel growth so that grains are split into many smaller subgrains. The evolution of a typical twin band is captured inside pink circles. For the 11 nm sample, the main deformation is grain boundary sliding and twin formation and growth. Additionally, we also observed that grain boundaries have a tendency to expand with increasing strain during small-size deformation gradually. For a relatively large sample of 23 nm, the deformation process is roughly the same as for a small size sample but slightly different. As shown in Figure 4b, twins appear earlier, and the number of twins develops more.

In Figure 4, we also observed that phase transitions of U-10Mo alloys from ‘bcc to fcc (hcp)’ occur locally, which has been observed previously in the literature [35,50]. According to the CNA results, the largest fractions of fcc and hcp atoms in all samples are about 4% and 1.4%, respectively, so we only discussed the fcc phase transition further. Supplementary Figure S2 displays the variation in the fraction of fcc atoms with strain for *d* = 11 and 23 nm samples. For atomic structure transition, the presence of a large number of fcc atoms is transient. Specifically, the fcc atoms in the 23 nm sample begin to increase significantly at a strain of 0.03. At 0.07 strain, there are already a large number of fcc atoms in the sample, and subsequently, the fcc atoms begin to decrease until their proportion drops to approximately 1%. In order to provide more local space for their adjacent bcc atoms, some bcc atoms must convert to locally fcc atoms when a significant number of bcc atoms convert to amorphous atoms, thereby lowering the energy barrier for transforming bcc atoms to amorphous atoms [58]. When U-10Mo alloy atoms are subjected to a sufficiently high applied stress load, the energy-raising ‘bcc to fcc’ transformation process is possible. When other plastic deformation paths are blocked or inhibited, highly steady bcc structures can be forcefully changed to reduce local stress concentration. However, twinning shear is more likely to occur to form twinning when two parallel {112} twin planes occur, leading to the low-priority of the high-cost ‘bcc to fcc’ deformation route [59], which explains why the fcc phase transition gradually decreases after the appearance of twins. In addition, amorphous atoms have greater potential energies than other regions, and those energies rise with increasing strain, resulting in the expansion of twin bands during stretching, as shown in the pink circles in Figure 4.

**Figure 5:**
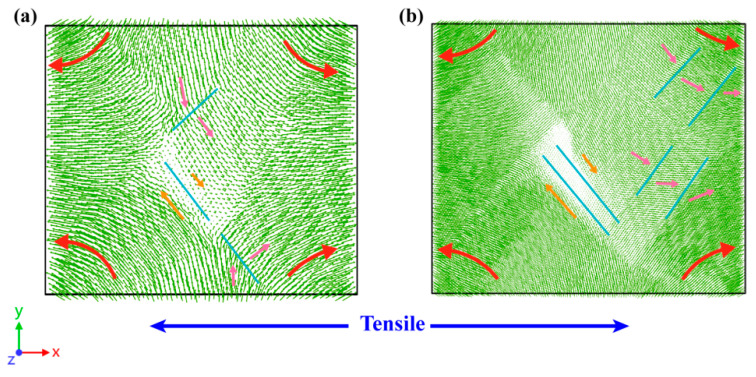
Snapshots of atomic displacement vectors at ε = 0.10 are shown in (**a**) 11 nm and (**b**) 23 nm, deformed at 300 K with strain rate of 5 × 10^8^ s^−1^.

Figure 5 illustrates atomic displacement vector diagrams of polycrystalline U-10Mo alloys with grain sizes of 11 and 23 nm at a strain level of 10%. The displacement vector for each atom points from its original position to its present location, and the movement trends of atoms are depicted by arrows drawn in various colors. The blue lines in Figure 5a,b represent grain boundaries and twin bands, respectively, with the red arrows indicating the overall movement trend of each grain. Different movement trends between the grains were captured, indicated by yellow and pink arrows, respectively. Grains spin and glide if the binding force between grains is lower than the shear stress, indicating the presence of grain rotation [51,60,61]. The atomic displacement vector can be used to identify such grain rotation, shown as red arrows in the diagram. In small-sized samples, grain rotation and grain boundary slipping were identified, which facilitates deformation. In contrast, for a sample with large grain size, grain rotation and grain boundary slipping are also present, yet it is twin bands growth and sliding that is the main deformation behavior, which likely results in a stress fall after achieving UTS (see Figure 2).

**Figure 6:**
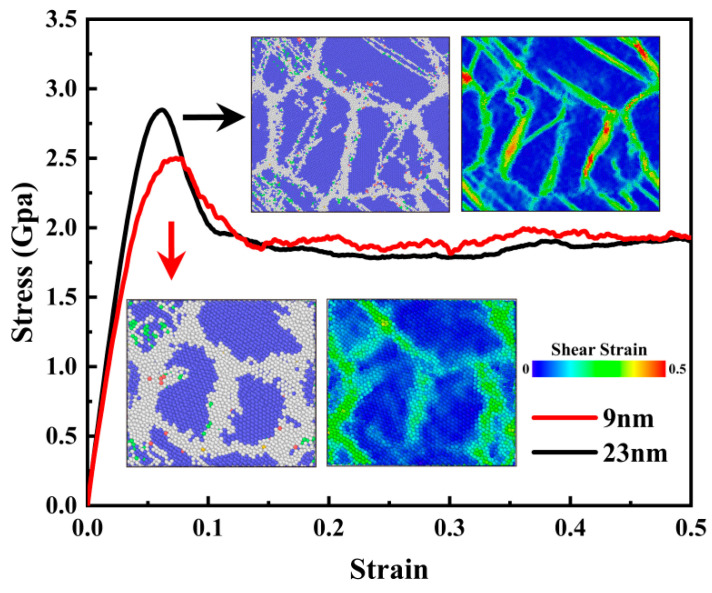
Uniaxial tensile stress–strain curves for samples with mean grain sizes of 9 and 23 nm. Snapshots of deformation processes at a strain of 10% subjected to CNA analysis and local strain analysis are provided in the insets.

In order to further explore the phenomenon that the greater the grain size, the more significant the stress reduction after achieving UTS, the stress–strain curve and local atomic deformation for *d* = 9 and 23 nm samples were contrasted, as shown in Figure 6. Obviously, a more significant stress drop is captured in the 23 nm sample after reaching the maximum value. By using the initial configuration before uniaxial stretching as a reference to quantify the localized atomic shear deformation, the following formula [50] was used to calculate the atomic von Mises shear strain, which was calculated from the six components of the atomic strain tensor:

(4) $$\eta_{i}^{Mises}=\sqrt{\eta_{yz}^{2}+\eta_{xz}^{2}+\eta_{xy}^{2}+\frac{{\left(\eta_{yy}-\eta_{zz}\right)}^{2}+{\left(\eta_{xx}-\eta_{zz}\right)}^{2}+{\left(\eta_{xx}-\eta_{yy}\right)}^{2}}{6}}$$

where $\eta_{i}^{Mises}$ is the von Mises shear strain per atom, and *η* is the Green–Lagrangian strain tensor calculated by the atomic strain module in OVITO. The region with relatively large $\eta_{i}^{Mises}$ value indicates that a large, localized plastic deformation of the atom has occurred [62]. The atomic deformation process at ε = 0.1 is depicted by the insets in Figure 6. Shear deformation is concentrated at grain boundaries for the 9 nm sample, whereas it is focused at grain boundaries and on twin bands for the 23 nm sample, with more severe shear strain occurring inside the twin bands. Stored strain energy can be released by the formation of twins in grains, resulting in sharp stress, which decreases behavior after reaching UTS. The nucleation of the twin starts from the grain boundary, in accordance with the observations in Figure 4. Therefore, it can be concluded that the coupling effect of grain boundary sliding, grain rotation and twins in grains contributes to the deformation process of NC U-10Mo alloys. For large-sized grains, the development of twins is the major deformation mechanism, while the deformation of small-sized grains is predominated by grain boundary sliding and grain rotation.

**Figure 7:**
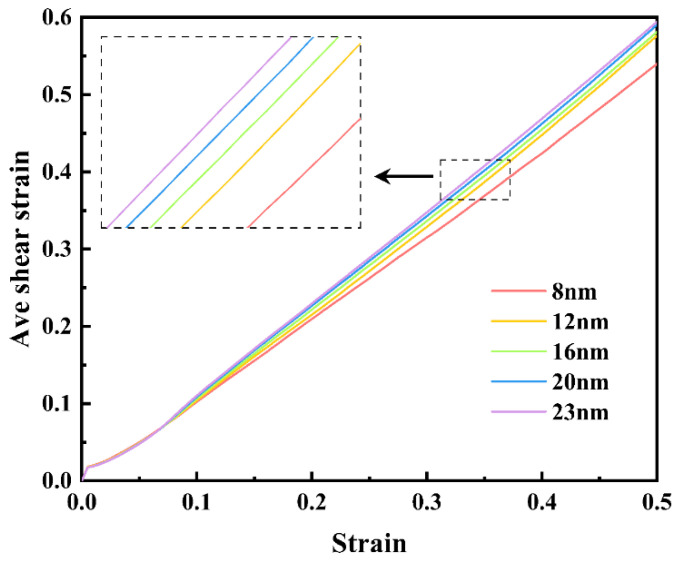
Evolutions of average atomic von Mises shear strain with tensile strain for polycrystalline U-10Mo alloys with different grain sizes of 8, 12, 16, 20, and 23 nm.

Furthermore, for all samples, the average von Mises shear strain was computed, and for brevity, the correlation between average shear strain and stretching strain for five typical samples is presented in Figure 7. After reaching UTS, samples with larger grain sizes show greater average shear strain values at the same strain, which means there is more shear deformation. Hence, it can be concluded that samples with larger grain sizes suffer from more severe local plastic deformation, which drives down the stress, resulting in a greater stress drop following peak stress.

### 3.3. Effect of Temperature

**Figure 8:**
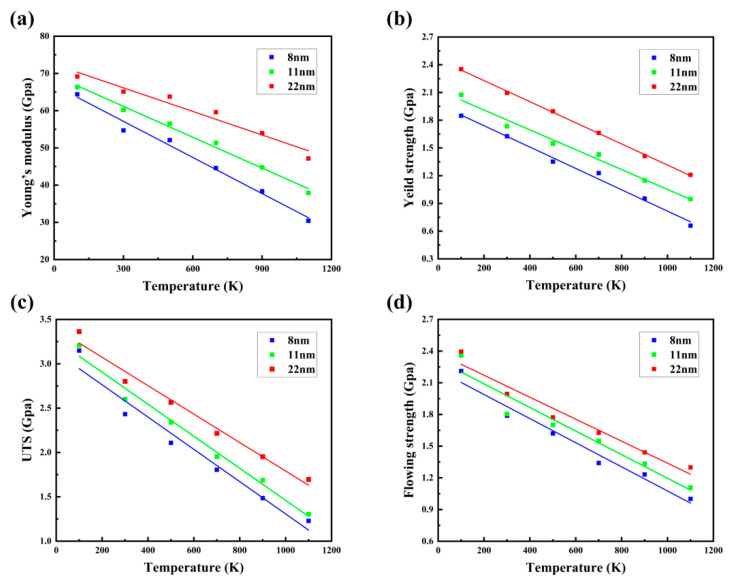
Influences of temperature on the mechanical properties of polycrystalline U-10Mo alloys with average grain sizes of 8, 11 and 22 nm: (**a**) Young’s modulus; (**b**) yield strength; (**c**) UST; (**d**) flow stress.

In this section, temperature dependency of the mechanical performance and deformation mechanisms of NC U-10Mo alloys is explored, which is also of interest. Tensile stress–strain curves for *d* = 22 nm sample at six various temperatures are displayed in Figure S3 in the Supplementary Materials. Six curves were observed exhibiting extremely similar tensile behaviors. However, its mechanical properties fluctuate with temperature. Figure 8a–d describe the evolution of the four mechanical parameters with temperature for *d* = 8, 11 and 22 nm samples, respectively. The flow stress *σ*_f_ is determined as a mean stress over the strain range 0.2–0.5. As can be seen from the diagram, four mechanical parameters appear to be linearly related to temperature, decreasing with increasing temperature. For instance, in the case of an 11 nm sample, Young’s modulus, yield strength, UTS and flow stress all fall when the temperature rises from 100 to 1100 K, increasing from 66.35, 2.08, 3.21 and 2.36 GPa to 37.92, 0.95, 1.30 and 1.11 GPa, respectively. We can conclude that high temperatures weaken the mechanical performance of NC U-10Mo alloys. The diffusion ability of atoms enhances, and the bond energies between the atoms decrease as the temperature rises, allowing the atoms to move more easily under external loads [63,64]. Consequently, high temperature will reduce the deformation resistance of the atoms, resulting in a lower Young’s modulus and strength.

**Figure 9:**
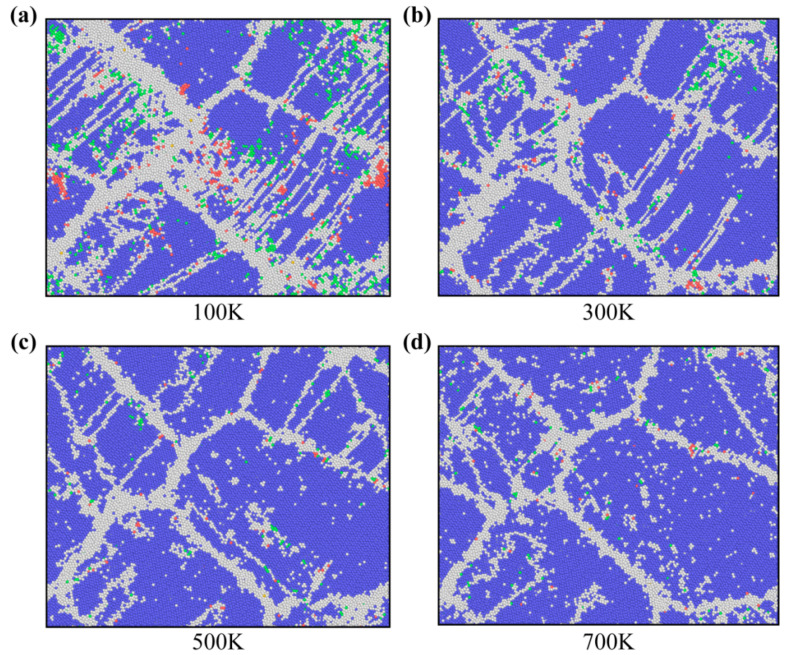
Snapshots of atomic configurations of NC U-10Mo alloys with a mean grain size of 22 nm at strains of 15%, deformed at temperatures of (**a**) 100 K, (**b**) 300 K, (**c**) 500 K and (**d**) 700 K. Atoms are colored according to the structure determined by CNA. Blue, green, red and white atoms correspond to BCC, FCC, HCP and other structures, respectively.

For plastic deformation of NC U-10Mo alloys, an important control parameter is temperature, which deserves further investigation. To assess the dependence of the twinning trend on temperature, Figure 9 compares the atomic structures of the samples at four temperatures when the strain is 15%. Suppression of twins in the sample was observed with increasing working temperature. Lower flow stresses may be responsible for the reduced twinning tendency at higher temperatures [26]. Supplementary Figure S4 shows the evolution of the fraction of grain boundary atoms with strain for samples with grain size *d* = 22 nm at different temperatures. We observed that the fraction of grain boundary atoms rises with decreasing temperature during the plastic deformation phase, which provides further evidence that low temperatures increase the twinning tendency. According to Zhao et al. [65], nanostructured Cu does not deform through twins at ambient temperature, while many deformation twins are generated at liquid nitrogen temperature, confirming that low temperature does facilitate twins in nanomaterials.

**Figure 10:**
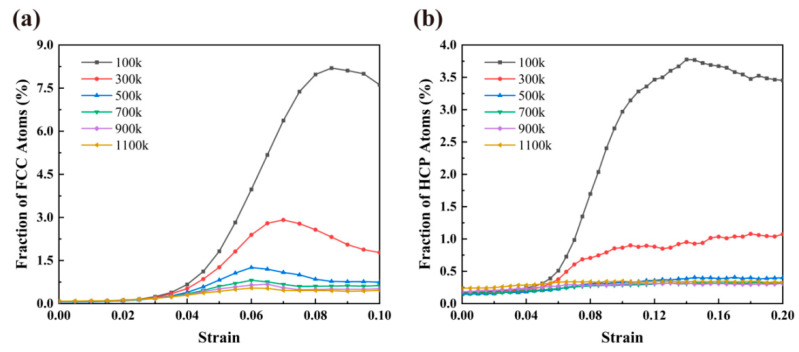
Fractions of fcc atoms (**a**) and hcp atoms (**b**) as a function of strain under various working temperatures of NC U-10Mo alloys with grain size of 22 nm. The applied strain rate is 5 × 10^8^ s^−1^.

We tracked the development of fcc and hcp atoms during the tensile process for the *d* = 22 nm sample at various temperatures, illustrated in Figure 10a,b. Compared to hcp atoms, fcc atoms at identical temperatures have a much higher maximum fraction. Notably, obvious phase transitions only occur at room temperature and below, owing to the fact that at higher temperatures, thermal activation allows the atoms to rearrange themselves to accommodate plastic strain without the need for phase transformations. However, this process is thermally forbidden at lower temperatures, so the rearrangement of atoms becomes difficult, and the probability of phase transition becomes higher, which may mean that the phase transition observed at lower temperatures is essentially displacive (diffusionless) due to the lower atomic mobility [66].

### 3.4. Effect of Strain Rate

**Figure 11:**
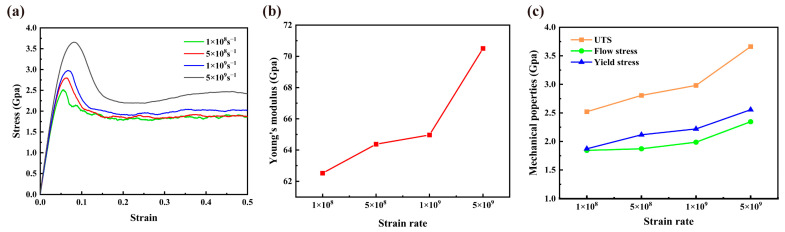
Influence of strain rate on the tensile behaviors of polycrystalline U-10Mo alloys with a grain size of 20 nm at 300 K: (**a**) Comparison of stress–strain curves at different strain loading rates of 1 × 10^8^, 5 × 10^8^, 1 × 10^9^ and 5 × 10^9^ s^−1^. (**b**) Variation in Young’s modulus with strain rate. (**c**) Variations in yield strength, UST and flow stress with strain rate.

The mechanical properties of nanomaterials were also shown to be significantly influenced by strain rate [67,68,69,70], so it is of guiding significance to research the mechanical properties of NC U-10Mo alloys at different strain rates before drawing conclusions. NC U-10Mo alloy with *d* = 20 nm was chosen to investigate the impact of strain rates, which were set to 1 × 10^8^, 5 × 10^8^, 1 × 10^9^ and 5 × 10^9^ s^−1^, respectively. As demonstrated in Figure 11a, analogous tensile behavior was observed under different strain rates. As a result of relaxation at lower strain rates, a slightly serrated behavior was observed in the plastic flow stage of the sample with a strain rate of 1 × 10^8^ s^−1^. During the elastic deformation phase, as strain rates rise, nanocrystalline U-10Mo alloys can withstand greater stress loads and show improved strength. Figure 11b,c give the values of Young’s modulus, UTS, flow stress and yield strength under four strain rates. A positive correlation between the mechanical parameters and strain rate was observed, similar to previous studies [50,56,67]. This was attributed to the fact that it was more difficult for the samples to fully release energy at higher strain rates, leading to enhanced mechanical properties.

**Figure 12:**
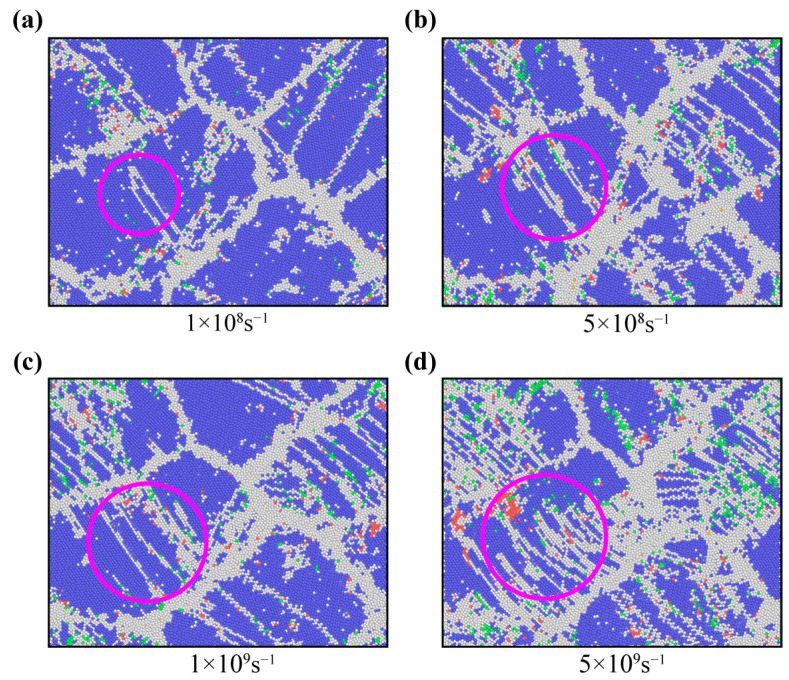
Snapshots of atomic configurations of NC U-10Mo alloys with a mean grain size of 20 nm at strains of 17.5%, deformed at strain rates of (**a**) 1 × 10^8^, (**b**) 5 × 10^8^, (**c**) 1 × 10^9^ and (**d**) 5 × 10^9^ s^−1^. Atoms are colored according to the structure determined by CNA. Blue, green, red and white atoms correspond to BCC, FCC, HCP and other structures, respectively.

To explore how strain rate affects the deformation of nanocrystalline U-10Mo alloys, Figure 12 compares the atomic structure of the *d* = 20 sample at four strain rates when the strain is 17.5% to evaluate the dependency of twinning tendency on strain rate. As observed in the graph, the formation of twins is facilitated by a high strain rate, in accordance with experimental results [27,71]. For example, comparing the twin trends in the pink circles in Figure 12, we can see that the number of twins at a 5 × 10^9^ s^−1^ strain rate is much more than that at 1 × 10^8^ s^−1^ strain rate. The rise in flow stress is primarily responsible for the increase in twinning tendency at lower temperatures and higher strain rates [26].

**Figure 13:**
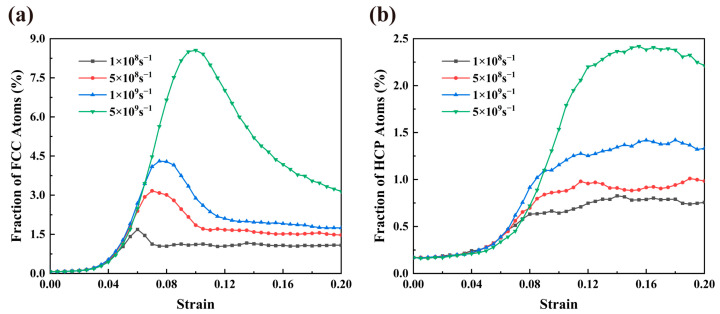
Fractions of fcc atoms (**a**) and hcp atoms (**b**) as a function of strain under various strain rates of NC U-10Mo alloys with grain size of 20 nm. The deformation was performed at 300 K.

Figure 13a,b depict the dependence of the fraction of fcc and hcp atoms on strain for four distinct stain rates, respectively. It is observed that for the same strain rate, the number of atoms undergoing the fcc phase transition far exceeds that undergoing the hcp phase transition. Furthermore, we find that increasing the strain rate improves the fcc and hcp atomic fraction maximum and delays the fcc atomic fraction maximum to larger strains significantly. This is reasonable because a too-large strain rate will make atoms significantly deviate from their original positions and make it difficult to return to the equilibrium positions, resulting in lattice distortion and causing more phase transitions.

## 4. Conclusions

In this study, MD simulation was used to carry out uniaxial tensile tests on nanocrystalline U-10Mo alloys with average grain sizes ranging from 8 to 23 nm. How the mechanical characteristics and deformation processes were affected by the average grain size was investigated. Moreover, the influences of temperature and strain rate on tensile behavior were also discussed. The key findings of this paper are as follows:

(1) Mechanical properties (including Young’s modulus, yield strength and UTS) monotonically improved as increasing average grain size. The inherent reason for Young’s modulus increasing with mean grain size was that atoms located in grains occupied a larger proportion.
(2) During deformation, localized phase transitions from bcc to fcc (hcp) were observed in polycrystalline U-10Mo alloys. Grains will spin and glide if the binding force between grains is lower than the shear stress. The coupling effect of grain boundary sliding and grain rotation as well as the development of twinning, contributed to the deformation process. The former is the main deformation mechanism in small-sized grains, while the deformation of large-sized grains is predominated by the latter.
(3) For larger-sized samples, the local plastic deformation was more serious, which promoted the decrease in stress, leading to a more obvious stress reduction after reaching the peak.
(4) Rising temperature weakened the mechanical properties of polycrystalline U-10Mo alloys while increasing the tensile strain rate caused the opposite. Lower temperatures and higher strain rates not only facilitated the twinning tendency but also favored the occurrence of phase transitions in samples.

Notably, the number of grains used in this investigation was insufficient to obtain valid statistics on the NC U-10Mo alloys, such that these conclusions should be tested using a system containing more grains in future simulations. Our simulations and detailed discussions will contribute to a greater comprehension of mechanical behavior and deformation mechanisms of polycrystalline U-10Mo alloys, which can help to determine their deformation and damage behavior under different stress and temperature conditions, thereby assessing their reliability and durability in various engineering applications. Moreover, understanding their microstructure will allow the development of new alloy design and preparation methods to improve their strength, plasticity and toughness, thereby expanding their application scope and effectiveness.

## Data Availability

The data that support the findings of this work are available from the corresponding author upon reasonable request.

## Supplementary Materials

The following supporting information can be downloaded at: https://www.mdpi.com/article/10.3390/ma16134618/s1, Figure S1: Uniaxial tensile stress–strain curves of nanocrystalline U-10Mo alloys with various grain sizes of (a) 8–15 nm and (b) 16–23 nm; Figure S2: Fraction of fcc atoms in NC U-10Mo alloys with the mean grain size of 11 nm and 23 nm at strains ranging from 0% to 10%, deformed at 300 K and a strain rate of 5 × 10^8^ s^−1^; Figure S3: Tensile stress–strain curves for NC U-10Mo alloys with a grain size of 22 nm for different temperatures at a strain rate of 5 × 10^8^ s^−1^; Figure S4: The evolution of the fraction of grain boundary atoms with strain for samples with grain size *d* = 22 nm at different temperatures; Table S1: Mechanical properties of nanocrystalline U-10Mo alloys with different grain sizes at 300 K and the strain rate of 5 × 10^8^ s^−1^.
