# Supplementary material for: Mechanical Properties and Deformation Mechanisms of Nanocrystalline U-10Mo Alloys by Molecular Dynamics Simulation
Source: Materials (Basel). 2023 Jun 27;16(13):4618. doi: 10.3390/ma16134618 (PMC10342338; doi:10.3390/ma16134618)
Supplement: Supplementary file 1 [file materials-16-04618-s001.zip › materials-2412368-supplementary.pdf]

materials

MDPI

Supplementary Material

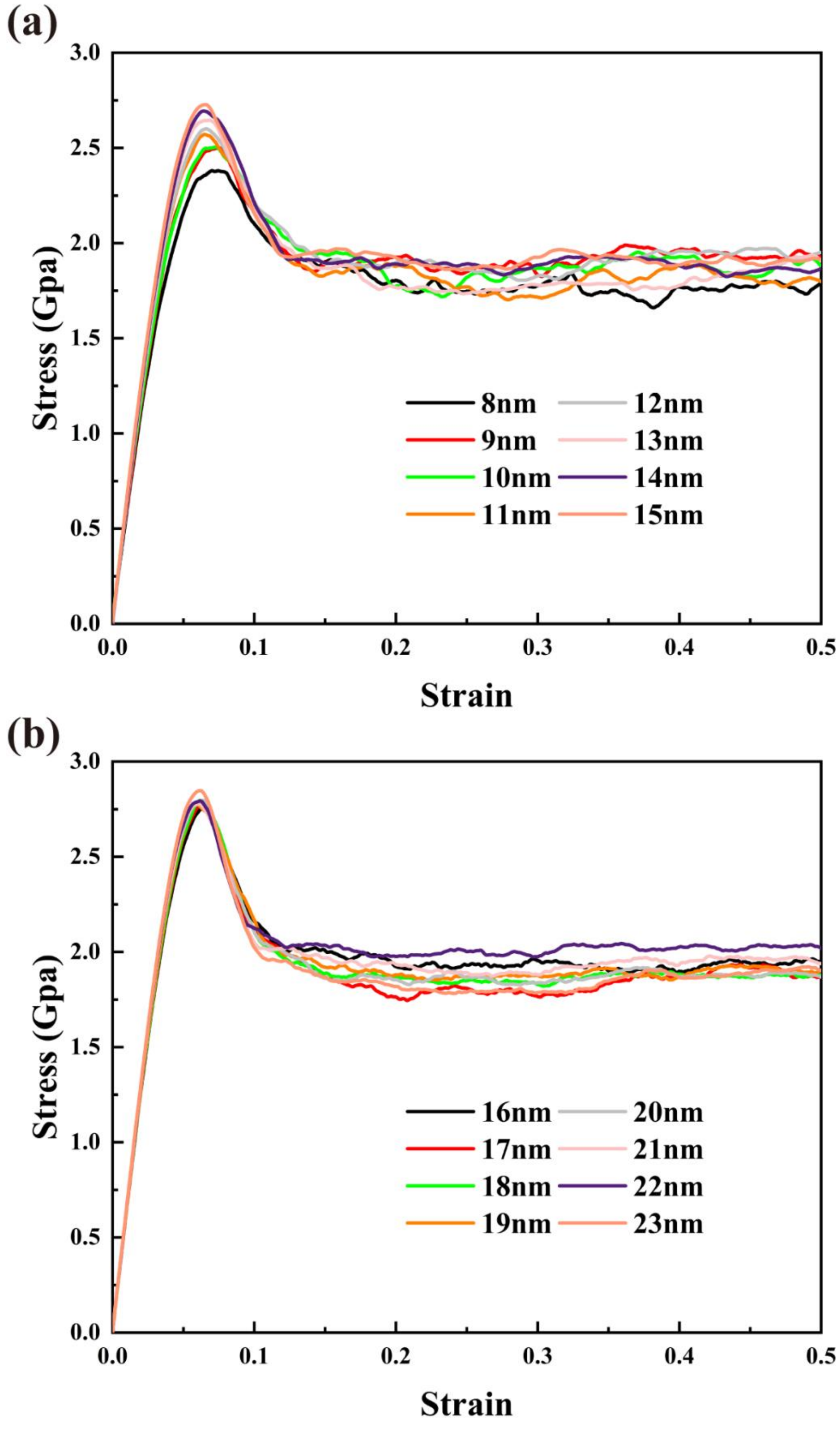

Figure S1. Uniaxial tensile stress-strain curves of nanocrystalline U-10Mo alloys with various grain sizes of **(a)** 8 - 15 nm and **(b)** 16 - 23 nm.

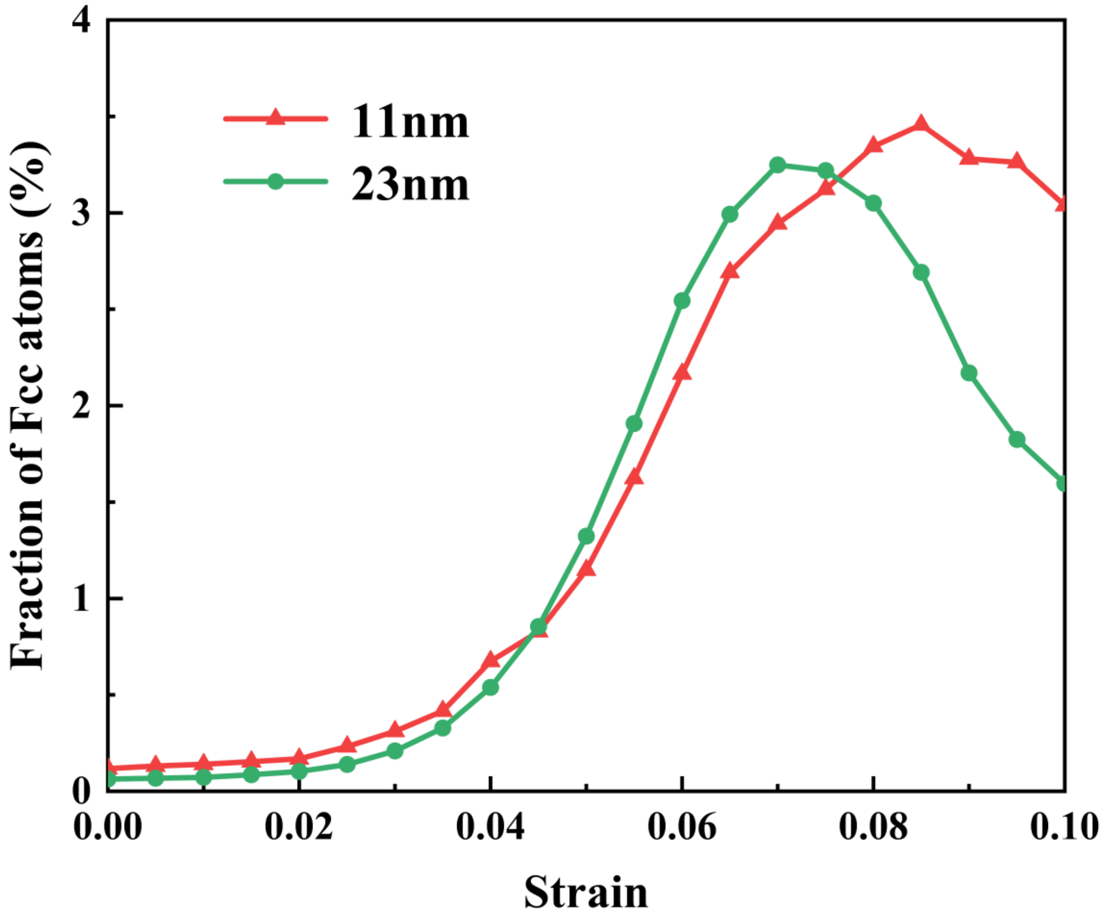

Figure S2. Fraction of fcc atoms in NC U-10Mo alloys with the mean grain size of 11 nm and 23 nm at strains ranging from 0% to 10%, deformed at 300 K and a strain rate of 5×10^{8} s^{-1}.

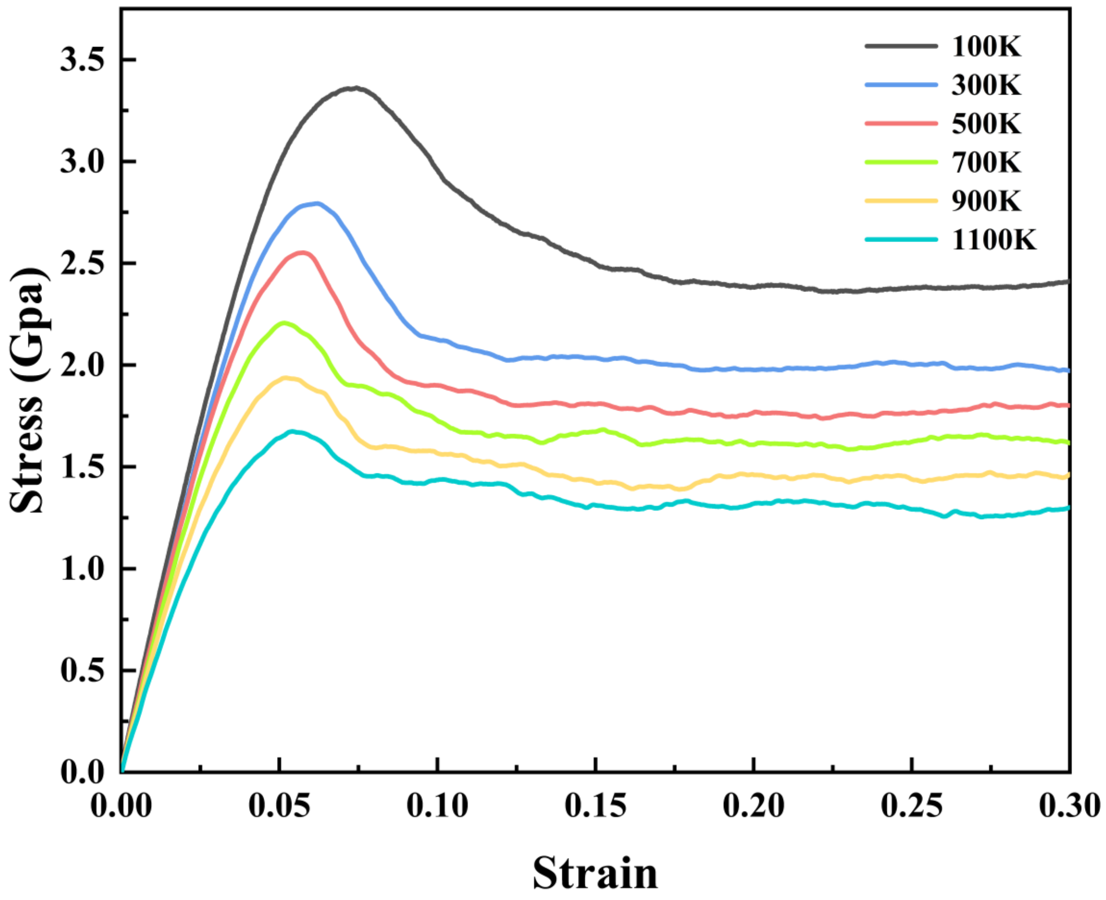

Figure S3. Tensile stress-strain curves for NC U-10Mo alloys with a grain size of 22 nm for different temperatures at a strain rate of 5×10^{8} s^{-1}.

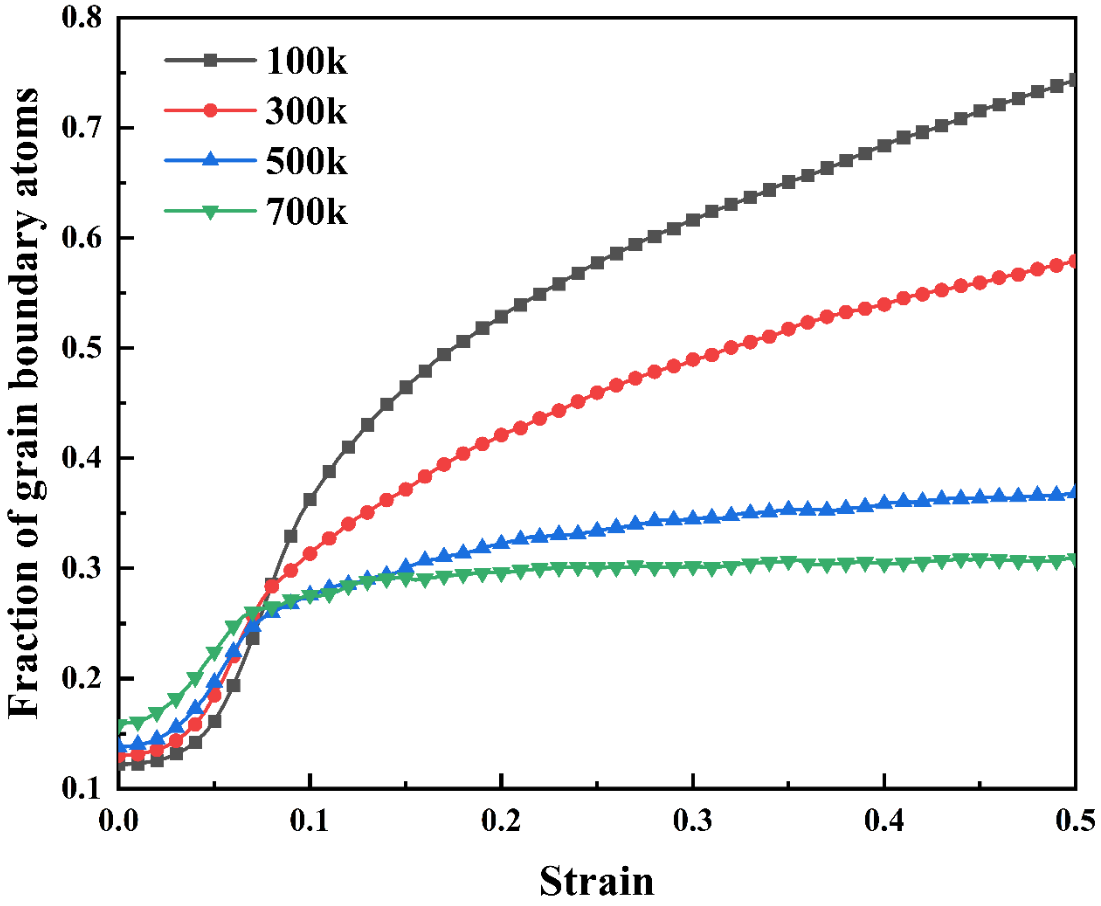

Figure S4. The evolution of the fraction of grain boundary atoms with strain for samples with grain size *d* = 22 nm at different temperatures.

Table S1. Mechanical properties of nanocrystalline U-10Mo alloys with different grain sizes at 300 K and the strain rate of 5×10^{8} s^{-1}.

Average grain size *d* (nm)	Lenth of sample *L* (nm)	Number of atoms *N*	Young's modulus *E* (GPa)	Yield strength σ_s (GPa)	UTS σ_b (GPa)
8	11.72	67,770	54.71	1.64	3.06
9	13.18	97,354	56.33	1.73	3.20
10	14.65	134,562	56.99	1.76	3.35
11	16.11	180,142	60.20	1.73	3.52
12	17.58	234,963	60.29	1.76	3.67
13	19.04	299,972	61.54	1.88	3.81
14	20.50	376,060	61.27	1.88	3.83
15	21.97	463,916	62.62	1.91	3.91
16	23.43	564,636	62.93	1.96	4.04
17	24.90	678,870	63.41	1.98	4.00
18	26.36	807,584	63.82	1.98	4.01
19	27.83	951,400	64.12	2.02	4.08
20	29.29	1,111,632	64.47	2.11	4.18
21	30.76	1,288,883	64.47	2.12	4.20
22	32.22	1,483,861	65.13	2.10	4.19
23	33.69	1,697,944	65.25	2.13	4.20
